# Inferior Shoulder Dislocation: A Report of Three Cases Reduced Using the ‘Two-Step Manoeuvre’

**DOI:** 10.7759/cureus.68982

**Published:** 2024-09-09

**Authors:** Jerry Sam, Aakaash Venkatesan, Rahul H Shah, Satish R Rohra, Leon Francis

**Affiliations:** 1 Orthopaedics and Traumatology, Aneurin Bevan University Health Board, Newport, GBR

**Keywords:** inferior shoulder dislocation, luxatio erecta, reduction, sub-coracoid, sub-glenoid, two-step manoeuvre

## Abstract

Inferior shoulder dislocations are uncommon, accounting for a very small percentage of all shoulder dislocations. A reduction technique has been developed which involves a two-step process: the first involves converting the inferior dislocation into an anterior one and the second involves reducing the humeral head back into its anatomical position within the glenohumeral joint. Traditional methods, such as the overhead traction-counter traction technique, often require multiple attempts, the involvement of several medical professionals, and the use of significant sedation and analgesia, which can be more intensive for the patient. Inferior dislocations that are positioned beneath the coracoid process present a particular challenge. Although they might seem suitable for reduction using methods typically applied for anterior dislocations, their positioning is often too inferior for these techniques to be effective. This two-step reduction technique has been utilised successfully in sub-coracoid and sub-glenoid inferior shoulder dislocations. The method has proven advantageous as a single practitioner can perform it, usually requires only one attempt, involves minimal force, and can be done under conscious sedation. These benefits make it a valuable alternative to traditional approaches for reducing inferior shoulder dislocations.

## Introduction

Inferior shoulder dislocations, also referred to as luxatio erecta, are an uncommon type of shoulder injury, making up only about 0.5% of all shoulder dislocations [1]. This condition is typically characterised by the arm being stuck in a hyper-abducted position, with the humeral head displaced beneath the glenoid fossa. The rarity of these injuries and their distinctive presentation can pose significant challenges for medical management. Most cases documented in the literature are linked to high-energy trauma incidents, such as falls from significant heights, motor vehicle accidents, or sports-related injuries [2].

Traditional techniques for reducing inferior shoulder dislocations, like the traction-counter traction method, often face difficulties achieving a successful reduction, even under deep sedation or anaesthesia [3]. These methods typically require multiple attempts and considerable force, raising the risk of complications and necessitating the involvement of multiple healthcare professionals. An alternative technique, the scapulohumeral manoeuvre described by Sonanis et al., involves redirecting the glenoid and humeral head towards one another; however, this approach also requires substantial manipulation and often an assistant [3].

There is a growing need for more efficient and less force-intensive techniques to minimise risks associated with conventional methods. In 2006, Nho et al. introduced the 'two-step manoeuvre' as an innovative approach for managing complex cases of inferior dislocations [4]. This method first converts the inferior dislocation to an anterior dislocation, which is then reduced into the glenohumeral joint using standard procedures for anterior dislocation. This approach is particularly beneficial as it typically requires only one practitioner, involves minimal force, and can be conducted under conscious sedation, making it a favourable alternative to traditional techniques [4].

Inferior shoulder dislocations are further categorised based on radiographic findings into sub-coracoid and sub-glenoid types [5]. Sub-coracoid dislocations are classified as inferior due to the humeral head's initial position below the glenoid and the coracoid. The term 'inferior' describes the humeral head's positioning in relation to the scapula, not necessarily in the strict vertical sense. The key differentiating features from an anterior dislocation are the degree of abduction and how the humeral head faces inferiorly instead of facing the glenoid. Despite sub-coracoid dislocations appearing to be amenable to anterior reduction techniques, they are often too inferior to be effectively managed by such methods alone [5]. While CT can provide detailed information about dislocation and associated injuries, it is typically reserved for cases with complex fractures or when initial reduction attempts fail. Plain radiographs usually suffice in most cases. Neurovascular examination must be undertaken before and after reduction as literature reports more than 50% incidence of neurological injury, although vascular injury is rare [1,4].

This report discusses three cases of inferior shoulder dislocations, two sub-coracoid and one sub-glenoid, successfully managed using the 'two-step manoeuvre' under minimal conscious sedation, without a change in post-reduction neurovascular status. Our experience highlights the effectiveness of this technique, especially in cases involving associated fractures, such as greater tuberosity fractures, which occur in approximately 31% of inferior dislocations [6].

## Case presentation

Two-step manoeuvre

**Figure 1:**
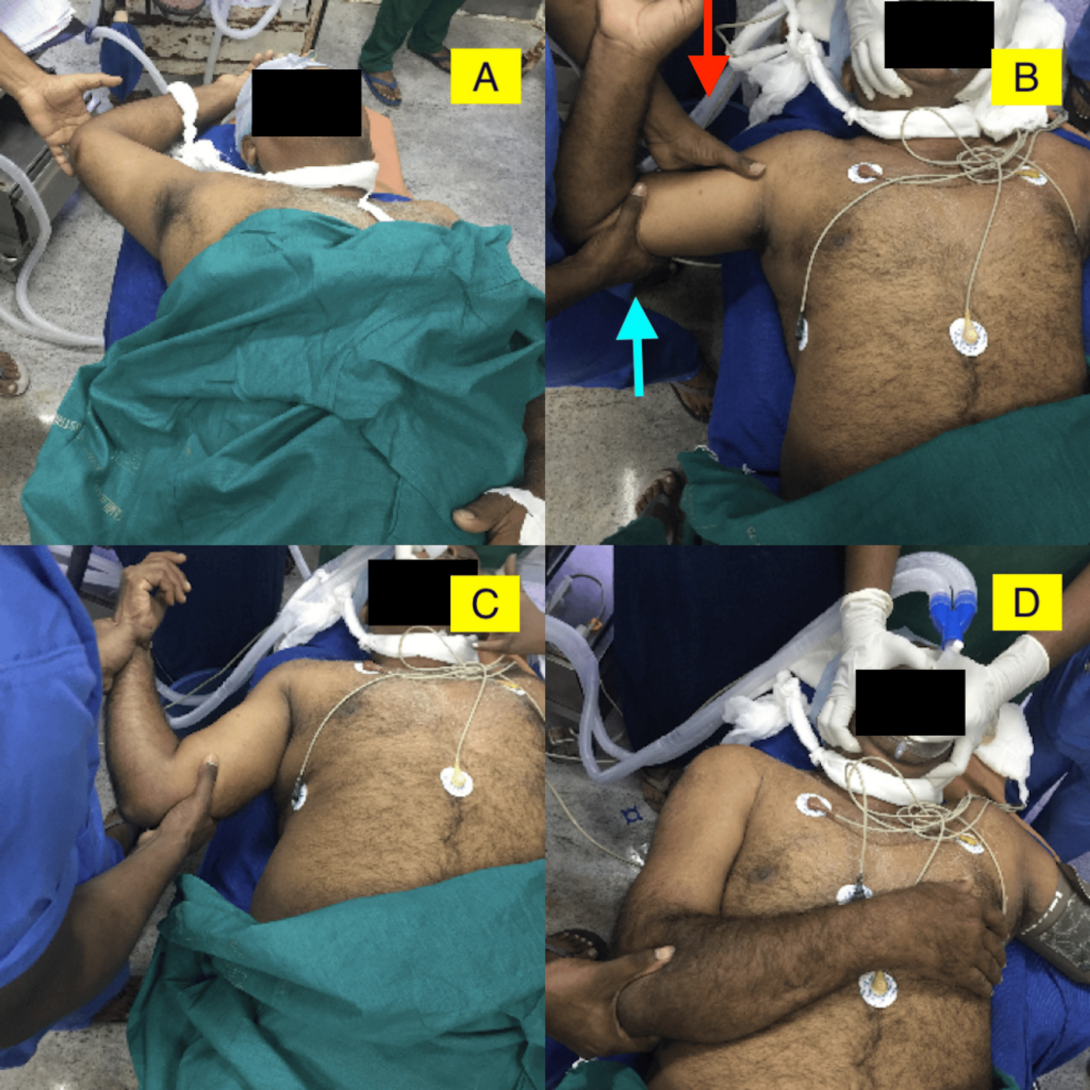
Steps of the 'two-step manoeuvre' A) The right arm is hyper-abducted with inferior shoulder dislocation. B) The author's left hand is used as a 'PUSH' hand (red arrow), and the right hand is used as a 'PULL' hand (blue arrow) to convert the inferior dislocation to an anterior one. Please look at the preceding paragraph for a more detailed explanation of the 'two-step manoeuvre'. C) The anterior dislocation is now reduced by the external rotation method. D) The arm and shoulder are resting in a reduced position.

The patient is positioned in a supine position with adequate sedation and analgesia. The surgeon positions his PUSH Hand on the lateral aspect of the mid-shaft of the humerus, manipulating the head to an anterior position. The PULL Hand, placed on the medial condyle of the distal humerus, provides traction and a gentle superior force directed at the distal humerus. The endpoint of this step is evidenced by the ability to adduct the humerus against the body, a prominent posterolateral border of the acromion and a straight outline of the shoulder. Once the head is anterior, the external rotation method is used for reduction into the glenohumeral cavity. (see Figure 1).

Case 1

A 20-year-old male was playing basketball when he was tapped on the arm while attempting to shoot with his shoulder in a hyperextended position. The patient was brought to the hospital with his right arm in an abducted position and had pain on any attempted movements. The patient’s arm was in 70 degrees of abduction, and the elbow was flexed to 90 degrees. The neurovascular examination was normal.

**Figure 2:**
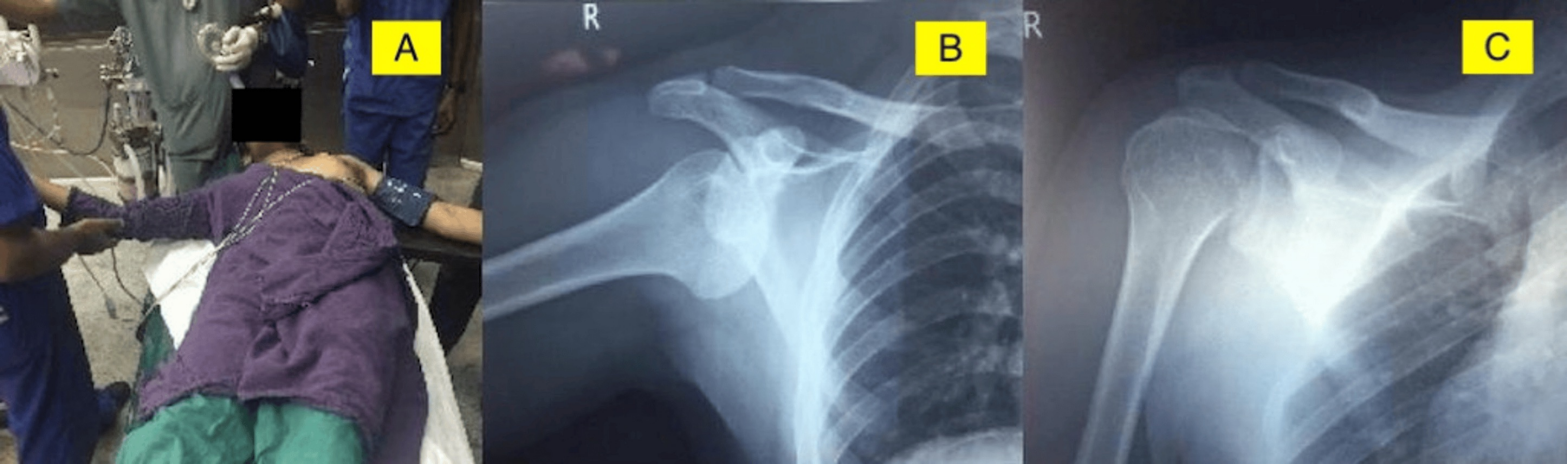
Case 1 A) The right arm and shoulder are in an abducted position with inferior shoulder dislocation. B) Radiograph showing right sub-coracoid inferior shoulder dislocation. C) Post-reduction radiograph showing shoulder in situ after 'two-step manoeuvre'.

Plain radiographs were obtained, and a sub-coracoid inferior shoulder dislocation was demonstrated. After manipulation, the patient had no neurovascular deficit, and the X-ray confirmed the reduction status and showed no bony defect in the humeral head. The patient was discharged in a shoulder immobiliser and followed up in one week (Figure 2).

Case 2

A 60-year-old male sustained injury to the right shoulder after a fall from a two-wheeler. The patient was initially treated elsewhere and gave a history of multiple failed attempts at reduction. The patient was brought to the hospital with his right arm in a hyper-abducted position and pain when attempting to move it. The arm was in 100 degrees of abduction, and the elbow flexed to 90 degrees. The neurovascular examination was normal.

**Figure 3:**
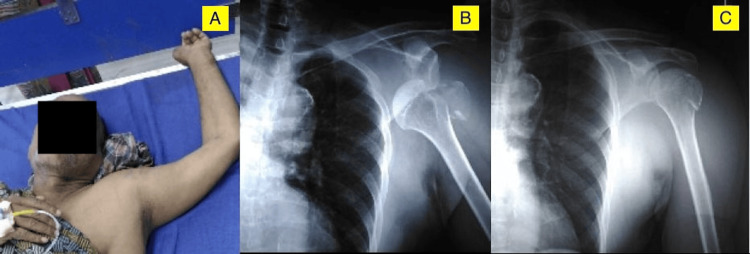
Case 2 A) The left arm and shoulder are in a hyper-abducted position with inferior shoulder fracture-dislocation. B) Radiograph showing left sub-coracoid inferior shoulder fracture-dislocation. C) Post-reduction radiograph showing shoulder in situ after 'two-step manoeuvre'.

Plain radiographs were obtained, and a sub-coracoid inferior shoulder dislocation with a greater tuberosity fracture was demonstrated. Post manipulation, the patient had no neurovascular deficit, and the X-ray confirmed the reduction status and showed no evidence of a bony defect in the humeral head. The patient was discharged in a shoulder immobiliser and was followed up in one week (Figure 3).

Case 3

A 52-year-old male sustained injury to the right shoulder after a fall from a two-wheeler. The patient was brought to the hospital with his right arm in a hyper-abducted position and had pain on any attempted movements. The patient’s arm was in 100 degrees of abduction, and the elbow was flexed to 90 degrees. The neurovascular examination was normal.

**Figure 4:**
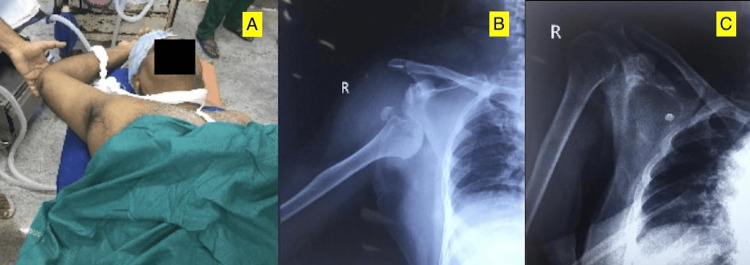
Case 3 A) The right arm and shoulder are in a hyper-abducted position with inferior shoulder fracture-dislocation. B) Radiograph showing right sub-glenoid inferior shoulder fracture-dislocation. C) Post-reduction radiograph showing shoulder in situ after 'two-step manoeuvre'.

Plain radiographs were obtained, and a sub-glenoid inferior shoulder dislocation with a greater tuberosity fracture was demonstrated. Post-manipulation, the patient had no neurovascular deficit. The X-ray confirmed the reduction status and showed no evidence of a bony defect in the humeral head. The patient was discharged in a shoulder immobiliser and followed up in one week (Figure 4).

## Discussion

Traumatic Inferior shoulder dislocation, luxatio erecta, is a rare type of shoulder injury, with only over 100 cases reported in English literature [2]. The reduction manoeuvres for inferior shoulder dislocations discussed in the literature include traditional methods like the traction-counter traction technique. This method, described historically, is often challenging due to the difficulty in achieving a successful reduction and the requirement for significant sedation and the use of strong narcotics to manage the associated pain and discomfort. The scapulohumeral manoeuvre is another method described by Sonanis et al. wherein the surgeon redirects the glenoid and humeral head towards each other with an assistant stabilising the limb [3]. The most characteristic feature is the hyper-abducted position of the arm at presentation, with the radiographic examination showing a subcoracoid or subglenoid dislocation. The inferior glenohumeral dislocation is further sub-classified into sub-coracoid and sub-glenoid varieties based on radiographic appearance [5]. Sub-coracoid dislocations are classified as inferior due to the humeral head's initial position below the glenoid and the coracoid. The term 'inferior' describes the humeral head's positioning in relation to the scapula, not necessarily in the strict vertical sense. The key differentiating features from an anterior dislocation are the degree of abduction and how the humeral head faces inferiorly instead of facing the glenoid. The sub-coracoid variety, although seemingly amenable to reduction by manoeuvres for anterior dislocation, in reality, is not as it is much inferior to be directly levered into the glenoid. We encountered two cases of sub-coracoid type and one sub-glenoid type, all reduced by the ‘two-step manoeuvre’.

Davids and Talbot described two mechanisms of injury in 1990 for luxatio erecta: direct and indirect [6]. In the direct mechanism, axillary loading on a fully abducted arm and the humeral head is driven through the weak inferior glenohumeral ligaments and joint capsule, frequently fracturing the greater tuberosity and tearing the rotator cuff. In the indirect mechanism, a violent abduction force on an already abducted limb levers the proximal shaft of the humerus over the acromion, and the humeral head rests below the glenoid in abduction. A careful neurovascular examination, both pre and post-reduction, is mandatory. The literature reports that more than half the cases have a concomitant Axillary nerve injury, although they have a good prognosis [1]. A vascular injury, although uncommon, has been reported. Although we did not encounter neurovascular deficits in our three cases, a high degree of suspicion is essential in each case.

Inferior dislocations are generally associated with surrounding soft-tissue injury. Although the diagnosis may be delayed, rotator cuff tears have been reported in 12% of cases. A recent literature review reported that 37% of inferior dislocations are associated with fractures of some type [1]. Greater tuberosity avulsion fractures occur in 31% of all cases, and there have been reports of fractures in the glenoid, acromion, surgical neck, humeral head, and scapular body. We encountered two cases with greater tuberosity fracture with a possible direct mechanism of injury. Occasionally, the humerus may buttonhole the inferior capsule, making the dislocation irreducible and requiring open reduction [7]. In a recent case report, arthroscopy before reduction of inferior glenohumeral dislocation demonstrated detachment of the superior labral anterior-posterior complex, which extended to the anteroinferior portion of the glenoid labrum [8].

The traction counter traction manoeuvre is the most commonly used closed reduction method requiring an assistant. With this overhead traction manoeuvre, the physician must provide a force that overwhelms all the muscles around the shoulder, which may be difficult even with conscious sedation. With the’ two-step manoeuvre', we reduced three cases with conscious sedation with a single attempt and a single operator. We noticed that the traction required on the PULL arm varies from case to case and consistently more in cases with greater tuberosity fracture, possibly due to the degree of soft tissue injury. However, long-term follow-up is essential to rule out additional injury due to the manoeuvre. The authors recommend using the 'two-step manoeuvre' for the primary reduction attempt based on experience with these three cases.

Limitations

We acknowledge that conclusions drawn from a small case series have inherent limitations. In the event of a failed reduction attempt, one must be prepared for open reduction as a fallback option with a high suspicion of button-holing. Long-term follow-up is essential to understand the outcomes and possible complications due to the procedure. Larger studies are needed to validate the findings further, but this may be a challenge in view of this being a rare injury.

## Conclusions

As inferior shoulder dislocations are rare, only a few reduction manoeuvres have been described in the literature. The use of the overhead traction-counter traction method may necessitate multiple attempts, at least two people, excessive sedation and analgesia. The ‘two-step manoeuvre’ is a closed reduction technique that first converts the inferior dislocation to an anterior dislocation, followed by closed reduction of the anterior dislocation, according to the surgeon's preference. This manoeuvre has been successful for our three cases and was performed with one operator, single reduction attempt, minimal force, under conscious sedation and without any neurovascular complications.

## References

[1] Mallon WJ, Bassett FH, Goldner RD (1990). Luxatio erecta: the inferior glenohumeral dislocation. J Orthop Trauma.

[2] Yamamoto T, Yoshiya S, Kurosaka M, Nagira K, Nabeshima Y (2003). Luxatio erecta (inferior dislocation of the shoulder): a report of 5 cases and a review of the literature. Am J Orthop.

[3] Sonanis SV, Das S, Deshmukh N (2002). A true traumatic inferior dislocation of shoulder. Injury.

[4] Nho SJ, Đodson CC, Bardzick KF (2006). The two-step maneuver for closed reduction of inferior glenohumeral dislocation (luxatio erecta to anterior dislocation to reduction). J Orthopaed Trauma.

[5] Sharma H, Denolf F (2004). Atypical subglenoid inferior glenohumeral dislocation clinically mimicking anterior dislocation. Eur J Trauma.

[6] Davids JR, Talbott RD (1990). Luxatio erecta humeri: a case report. Clin Orthop Relat Res.

[7] Davison BL, Orwin JF (1996). Open inferior glenohumeral dislocation. J Orthop Trauma.

[8] Schai P, Hintermann B (1998). Arthroscopic findings in luxatio erecta of the glenohumeral joint: case report and review of the literature. Clin J Sport Med.

